# Optimizing Mechanical Ventilation: A Clinical and Practical Bedside Method for the Identification and Management of Patient–Ventilator Asynchronies in Critical Care

**DOI:** 10.3390/jcm14010214

**Published:** 2025-01-02

**Authors:** Vasco Costa, José Pedro Cidade, Inês Medeiros, Pedro Póvoa

**Affiliations:** 1 Department of Critical Care Medicine, Hospital de São Francisco Xavier, Unidade Local de Saúde Lisboa Ocidental (ULSLO), Estrada Forte do Alto Duque, 1449-005 Lisbon, Portugal; zencidade@gmail.com (J.P.C.); imedeiros@ulslo.min-saude.pt (I.M.); pedrorpovoa@gmail.com (P.P.); 2 NOVA Medical School, New University of Lisbon, 1169-056 Lisbon, Portugal; 3 Center for Clinical Epidemiology and Research Unit of Clinical Epidemiology, OUH Odense University Hospital, DK-5230 Odense, Denmark

**Keywords:** patient–ventilator asynchronies, ventilator waveforms, respiratory monitoring, mechanical ventilation

## Abstract

The prompt identification and correction of patient–ventilator asynchronies (PVA) remain a cornerstone for ensuring the quality of respiratory failure treatment and the prevention of further injury to critically ill patients. These disruptions, whether due to over- or under-assistance, have a profound clinical impact not only on the respiratory mechanics and the mortality associated with mechanical ventilation but also on the patient’s cardiac output and hemodynamic profile. Strong evidence has demonstrated that these frequently occurring and often underdiagnosed events have significant prognostic value for mechanical ventilation outcomes and are strongly associated with prolonged ICU stays and hospital mortality. Halting the consequences of PVA relies on the correct identification and approach of its underlying causes. However, this often requires advanced knowledge of respiratory physiology and the evaluation of complex ventilator waveforms in patient–ventilator interactions, posing a challenge to intensive care practitioners, in particular, those less experienced. This review aims to outline the most frequent types of PVA and propose a clinical algorithm to provide physicians with a structured approach to assess, accurately diagnose, and correct PVA.

## 1. Introduction

The prompt identification and correction of patient–ventilator asynchronies (PVA) remain a cornerstone for ensuring the quality of respiratory failure treatment and prevention of further injury in critically ill patients [1,2]. The mismatch between patients’ ventilatory demands and the support provided by the ventilator represents a common phenomenon, with an incidence of up to 80% of patients at some point during the ICU length of stay [3]. A previous report found that one-quarter of mechanically ventilated ICU patients experience severe asynchrony, defined by a high frequency of asynchrony events normalized to total respiratory rate, including wasted efforts [4,5].

These disruptions—resulting from over- or under-assistance—have profound clinical consequences, namely respiratory mechanics, cardiac output, and hemodynamic stability. PVAs are often associated with increased work of breathing and ventilator-induced lung injury due to diaphragm–lung dissociation, excessive tidal volumes, and higher mechanical strain [6,7]. Furthermore, asynchronous patient efforts cause intrathoracic pressure fluctuations, which can disrupt ventricular Starling forces and adversely affect cardiac output and hemodynamics [8]. Furthermore, growing evidence has demonstrated that these frequently occurring and often undiagnosed events may significantly influence mechanical ventilation outcomes [9], potentially contributing to prolonged ICU stays and increased hospital mortality [10].

Halting the consequences of PVA relies on the precise identification and management of its underlying causes. Despite recent technological advances, including machine learning models [11], accurate detection often requires advanced knowledge of respiratory physiology and detailed analysis of ventilator waveforms, which remains challenging for many intensivists [12,13]. Therefore, a structured approach to PVA management is crucial to improve patient–ventilator synchrony, limit adverse effects, and enhance ventilation comfort and outcomes. This review aims to describe the most frequent types of PVAs and present a clinical algorithm that provides a structured approach for accurate PVA assessment, diagnosis, and correction.

## 2. Methods

### 2.1. Search Strategy

This narrative review was conducted to comprehensively summarize the existing literature on patient–ventilator asynchrony (PVA) and its management. A systematic search of the published literature was performed using the PubMed/MEDLINE, Scopus, and Web of Science databases. The following keywords and Medical Subject Headings (MeSH) terms were used for the search query: “patient-ventilator asynchrony”, “asynchrony management”, “waveforms”, “ventilator”, “bedside waveform interpretation”, “monitoring”, “mechanical ventilation”, and “patient-ventilator interaction”. A manual search of the reference lists of relevant studies and key review articles was also performed to identify the additional pertinent literature.

### 2.2. Inclusion and Exclusion Criteria

We included studies that fulfilled the following criteria: studies involving adult patients (≥18 years) undergoing invasive mechanical ventilation; studies written in the English language; studies focusing on patient–ventilator asynchrony, including its types, clinical impact, diagnostic methods, and management strategies; and studies discussing the use of ventilator waveforms, bedside waveform interpretation, monitoring tools, and strategies to optimize patient–ventilator interaction.

Exclusion criteria were as follows: studies focused exclusively on pediatric or neonatal populations; studies lacking original data or detailed discussion of PVA (e.g., commentaries, opinion pieces, and editorials); studies with full-text not accessible or not published in peer-reviewed journals and studies unrelated to the specific keywords or outside the scope of mechanical ventilation and PVA management.

### 2.3. Study Selection and Data Extraction

The titles and abstracts of retrieved articles were screened independently by two reviewers. Full-text articles meeting the inclusion criteria were then assessed for eligibility. Any discrepancies between reviewers were resolved through discussion or consultation with a third reviewer. Data were extracted using a standardized template, which included types of asynchronies, diagnostic tools, and management approaches.

### 2.4. Quality Assessment

To ensure methodological rigor, the quality of included studies was assessed using appropriate tools: the Cochrane Risk of Bias tool for randomized controlled trials and the Newcastle–Ottawa Scale for observational studies. For systematic reviews and meta-analyses, we evaluated quality using the AMSTAR-2 checklist. Articles with significant methodological limitations were not included.

### 2.5. Data Synthesis

Given the heterogeneity of study designs and outcomes, a narrative synthesis approach was adopted. The findings were categorized into themes, including types and pathophysiology of patient–ventilator asynchrony; diagnostic strategies, including waveform interpretation and monitoring tools; and strategies for PVA prevention and management, including algorithm-based approaches.

### 2.6. Ethical Considerations

This study did not involve human participants or interventions and, therefore, did not require ethical approval.

## 3. Results

**Chart 1:**
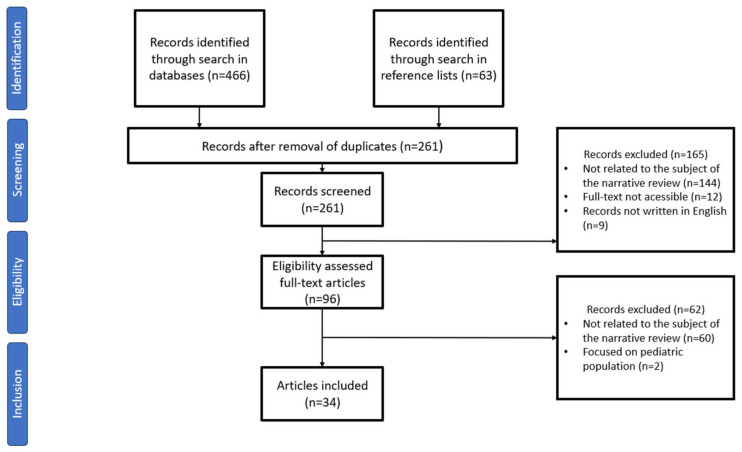
Flowchart showing results of the search strategy.

A total of 529 studies were identified through the database search and reference lists. After removing duplicates (268 studies), 261 studies were screened based on their titles and abstracts. Following this initial screening, 96 studies were deemed eligible for full-text narrative review. Of these, 34 studies met the inclusion criteria and were included in the final review, as depicted in Chart 1.

## 4. How to Identify Patient–Ventilator Asynchronies

When managing a ventilated patient, the ventilator alarms are often the first manifestation that the patient is unwell. Furthermore, the nurses’ care and comments are also a reliable source of information. Nevertheless, in a scenario where a patient–ventilator asynchrony is suspected, the physician’s frequent observation at the bedside is of utmost importance [14,15]. These periods of observation must include analyzing the patient and the ventilator screen [16] since both may provide invaluable information and quite often point the trained physician toward the correct asynchrony [15].

Firstly, the patient’s facial expression may suggest signs of discomfort, pain, or anxiety [17]; alongside this, the physician may ask basic and direct questions to exclude that any of these causes may contribute to developing PVAs. Coughing and hyperpnea are also frequent signs of PVAs. Touching the patient’s thorax may help to assess the consonance between the patient’s effort (assess muscle contraction), the programmed ventilator settings, and the delivered respiratory cycle.

Secondly, the careful analysis of the ventilator curves, particularly the pressure and flow curves [17,18], remains indispensable. This is not an easy task and requires specific training [15]; nonetheless, it is conveniently accessible, reproducible, and has been compared to more invasive techniques with good results [5]. Specific changes in the pressure and flow curves may steer towards singling out one or another type of PVA, namely the phase of the respiratory cycle they occur in, and if they resolve after ventilator or other therapeutic adjustment. These patterns will be discussed further in the following section.

Apart from the visual inspection of the ventilator waveforms, other techniques may be applied, including diaphragmatic ultrasound and an esophageal catheter [18]. The latter, albeit invasive and with additional costs for the disposable catheter and specific software, may contribute greatly by providing information regarding the patient’s inspiratory effort and, in other cases, identifying the particular type of PVA present by evaluating the positive or negative swings in esophageal pressure (Pes) and when these occur during the respiratory cycle [18,19,20].

The clinical impact of PVAs is clearly defined [19,21]. Inadequate interaction between the patient and the ventilator will cause the patient discomfort and dyspnea and lead to unnecessary and avoidable anxiety. Furthermore, the presence of PVA prolongs the weaning process [9], related to the wasted efforts in terms of energy expenditure that may prolong mechanical ventilation duration. Finally, in the case of particular types of PVAs, repetitive asynchronous respiratory muscle contractions against closed respiratory valves—eccentric contraction—may alter the muscles’ structure and, ultimately, function, thus resulting in an additional obstacle to successful weaning.

After establishing and identifying that the patient is presenting some type of PVA, the burden of the PVA may be roughly estimated by calculating the asynchrony index—number of asynchrony events/total respiratory rate, where the total respiratory rate includes the ventilator cycles and the number of ineffective triggers [5,19]. In various investigations, the asynchrony index value > 10% is applied to define the high incidence of asynchronies, and many have revealed a correlation with patient discomfort, longer mechanical duration, and mortality [22].

## 5. Classification of Asynchronies

The classification presented in Table 1 is a simplified way to organize the main types of asynchronies. In general, asynchronies are more frequently encountered when the patient presents some degree of respiratory drive or during the ventilator weaning phase. Thus, they usually occur in assisted modes of ventilation, specifically during pressure support or volume assist [9]. An exception to this is a recently described form of asynchrony, reverse triggering, which develops during controlled modes of mechanical ventilation.

In the subsequent sections, based on the present classification, nine types of asynchronies will be discussed in terms of definition, identification, etiology, and suggested management.

### 5.1. Asynchronies Definition, Identification, and Management

#### 5.1.1. Triggering

##### Inspiratory Trigger Delay

In this clinical scenario, there is a long time gap between the patient’s inspiratory effort and the beginning of the mechanical inspiratory cycle [23]. Careful observation and palpation of the patient’s upper abdomen to ascertain the timing of the beginning of the inspiratory effort while simultaneously observing the ventilator curves for the onset of the inspiratory flow will detect this time lag.

**Figure 1:**
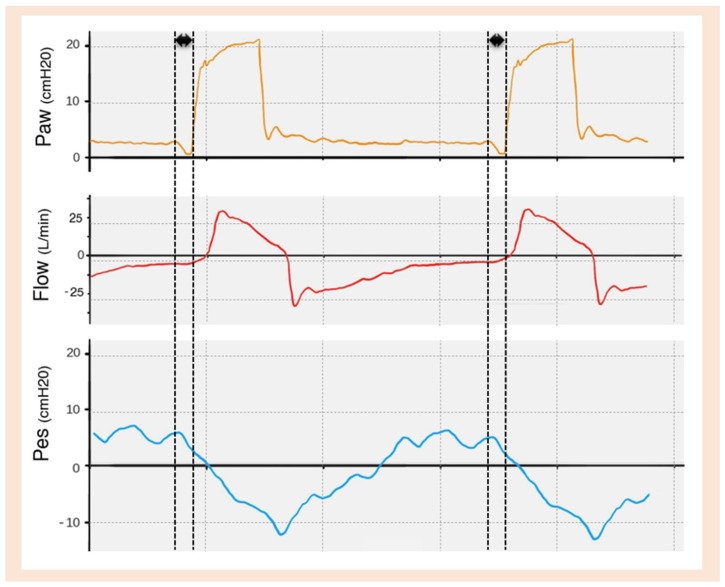
Inspiratory trigger delay—the beginning of the inspiratory effort is evident from the negative deflection in the pressure scalar that precedes the positive deflection corresponding to the delivered mechanical breath (two dashed lines). In this PVA, the delay between these two respiratory stages may be measured (double arrow = delay time). PVA: patient–ventilator asynchrony.

By observing the ventilator, when an inspiratory trigger delay is present, there will be an initial negative deflection in the pressure scalar (start of the patient’s inspiratory effort—see Pes tracing in Figure 1), followed only after an amount of time by a positive deflection both in the flow and pressure scalar curves [24], thus indicating an increase in patient’s airway pressure accompanied by the increase in inspiratory flow, as can be observed in Figure 1. Furthermore, the pressure curve graph allows for the measurement of this time lag in milliseconds (ms), being diagnostic of this VA if the interval is > 200 ms.

The main causes related to the development of inspiratory trigger delay are a leak in the ventilator circuit, a low-sensitive trigger, or cases where increased intrinsic positive end-expiratory pressure (PEEP) is present or programmed external PEEP is excessive [25]. A leak in the ventilator circuit will alter the connection between the patient’s airway via the tracheal tube and the ventilator circuit and machinery, thus hindering the ventilator’s ability to adequately respond to the patient’s initial respiratory effort.

Secondly, the reduced sensitivity of the inspiratory trigger simply implies that the ventilator will require a greater inspiratory effort from the patient to open the inspiratory valve and initiate the assisted mechanical breath, whether this effort is in terms of negative pressure or flow, which is sensed by the ventilator. This is also related to another trigger patient–ventilator asynchrony—ineffective trigger, discussed in the following section.

Another scenario is found in patients who, either from the causative disease that motivated invasive mechanical ventilation or from a past medical condition such as chronic obstructive pulmonary disease (COPD) or asthma, may have developed dynamic hyperinflation and subsequently autoPEEP. When present during the weaning phase, autoPEEP imposes an additional inspiratory pressure burden to be counterbalanced, apart from that already present from the inspiratory trigger settings. In this case, the patient will take longer to overcome this new threshold to achieve the minimal requirements for the ventilator to open the inspiratory valve. Similarly, this may also originate cases of ineffective triggering.

Management consists of altering the sensitivity of the inspiratory trigger to facilitate the initial workload of the patient and to review the ventilator–patient circuit (including the filters) for any possible leaks. Ultimately, when autoPEEP is present, adding or increasing the dose of bronchodilators and increasing the external PEEP to promote easier triggering may reduce or resolve this asynchrony.

##### Ineffective Trigger

Ineffective triggering, or ineffective inspiratory triggering, occurs when the patient’s inspiratory effort does not successfully generate the inspiratory phase of the mechanical breath by the ventilator [18,25]. This asynchrony is also known as “missed trigger”, “wasted effort”, or “failure to trigger” and is one of the most frequent PVAs observed in the ICU [5,9,26]. The patient generally is unaware of what is happening since it does not cause any discomfort [25,27]. By interviewing the patient, the physician may inquire whether the ventilator is providing a breath every time the patient requires one. Regarding physical examination, the physician may place a hand in the patient’s upper abdomen to attempt to perceive the diaphragmatic contraction and simultaneously listen to the ventilator to observe if it is followed by a mechanical breath [17,21].

**Figure 2:**
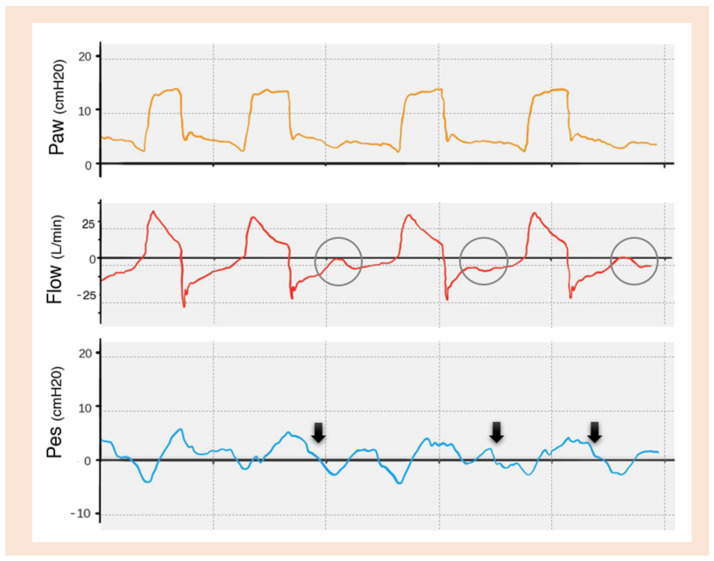
Ineffective trigger—only discrete alterations may be present, such as negative deflection in the pressure scalar or a positive deflection in the expiratory flow curve with no subsequent mechanical breath (circled areas). In this particular scenario, the simultaneous negative deflections depicted in the Pes curve indicate that these correspond to the patient’s active inspiratory, but there are unsuccessful efforts (arrows). Pes: esophageal pressure.

Regarding waveform manifestations, subtle changes may be apparent in the pressure curve with a negative deflection and a positive deflection in the expiratory flow curve [16,18,26,27], as per Figure 2. If this asynchrony is excessively severe, the patient’s breathing may become time-cycled (by the backup set respiratory rate). The relevance of using an esophageal catheter is unquestionable in this scenario, where the missed triggers will be effortlessly detected by negative deflections in the Pes curve without simultaneous initiation nor delivery of an assisted breath [20,27,28].

The main causes attributed to this phenomenon consist of an inadequate inspiratory trigger, autoPEEP, poorly programmed external PEEP, pressure over assistance by the ventilator, and diaphragmatic atrophy [4,18,21,25].

Firstly, the “sensitivity” of the trigger, whether programmed as a variable of pressure or flow, simply correlates to how efficiently and comfortably the patient may set off the ventilator to open the inspiratory valve and thus begin the mechanical breath. If the programmed inspiratory trigger is set at a very high value, this may become overstrenuous for the patient, thus impeding the activation of the ventilator and, ultimately, will not result in the initiation of a mechanical breath.

Secondly, scenarios of installed autoPEEP may lead to ineffective triggering [18,29]. This might arise from cases of dynamic hyperinflation in COPD, excessive tidal volumes, or increased respiratory rate with decreased time for expiration and other clinical pictures. In these situations, in order to activate the ventilator, the patient now must achieve an inspiratory effort that overcomes the added intrinsic pressure to the set trigger target. Excessive tidal volumes may be a direct consequence of pressure over assistance, which, in turn, contributes to the development of autoPEEP. Furthermore, poorly set external PEEP may contribute to such a scenario of ineffective triggering [30]. Moreover, patients with diaphragmatic atrophy (prolonged neuromuscular blockade and/or prolonged controlled mechanical ventilation) or dysfunction, as in certain neuromuscular diseases, may impair the patient’s ability to generate enough strength to trigger the ventilator.

Managing ineffective triggering may involve applying different strategies [16,21]. One of the simplest includes altering the sensitivity of the trigger, i.e., facilitating the patient’s activation of the mechanical breath [31]. This may be achieved by altering the setting of the trigger by reducing the amount of flow (L/min) or pressure (cmH2O) required to activate the ventilator. Reducing the depth of sedation and aiming at a target Richmond Agitation-Sedation Score (RASS) score of between −2 and 0 is another option to enhance the patient’s respiratory drive and thus ease effective triggering.

Furthermore, in cases where autoPEEP has been identified, this most certainly must be addressed, essentially by decreasing the increased airway resistance either by adequate endotracheal tube aspiration or by initiating or optimizing bronchodilator therapy. Other strategies to decrease autoPEEP would be to reduce respiratory rate (in assist/controlled ventilation modalities) and to increase expiratory time. In terms of excessive inspiratory pressure, the resolution is fairly straightforward by simply reducing the pressure value and evaluating whether the resulting tidal volumes are adequate [29] and if the autoPEEP has improved.

Lastly, the addition of extrinsic PEEP aids the weaning of patients with autoPEEP [31]. In these patients, if no external PEEP is applied, the inspiratory effort needed to trigger the ventilator is higher (from the value of autoPEEP present to the trigger threshold) [29]. In contrast, if external PEEP is set (below the autoPEEP present), then the inspiratory effort required will be less pronounced (the decrease in the amplitude of the effort is equal to the set external PEEP), leading to reduced work of breathing and less frequent ineffective inspiratory efforts. Nevertheless, as previously discussed, inadequately programmed external PEEP may lead to the worsening of dynamic hyperinflation. Thus, the external PEEP must be programmed below 75–85% of the measured autoPEEP [21,25].

##### Autotrigger

Autotrigger develops when the ventilator produces a mechanical breath without the previous effort from the patient; thus, it is an “unintentional” assistance by the machine [4,21]. This will lead to a mismatch between the patient’s respiratory rate and the one delivered by the ventilator. Autotriggering is more frequent in flow-trigger settings [25].

The main sources for this asynchrony include vigorous cardiac oscillations, leaks in the ventilator circuit or the presence of secretions/fluid in the circuit, hiccups, and chest drains with high negative pressure, among others [4,25]. All of these generate false changes in either flow/pressure, thereby achieving the ventilatory threshold for initiating a mechanical breath [18], generally to a baffled patient. In cases of suspected brain death, autotrigger may hinder the ability to initiate the neurological examination to determine this diagnosis by falsely providing the patient with spontaneous respiratory drive. This will lead to many negative consequences, namely for the patient but also for the family, the clinical team, and, finally, possible organ recipients.

The discrepancy between the patient’s requirements and what the ventilator is providing leads to an unexplained increased respiratory rate and also patient discomfort. The physician may approach the patient by enquiring whether the patient is receiving “unwanted” assisted breaths [23].

**Figure 3:**
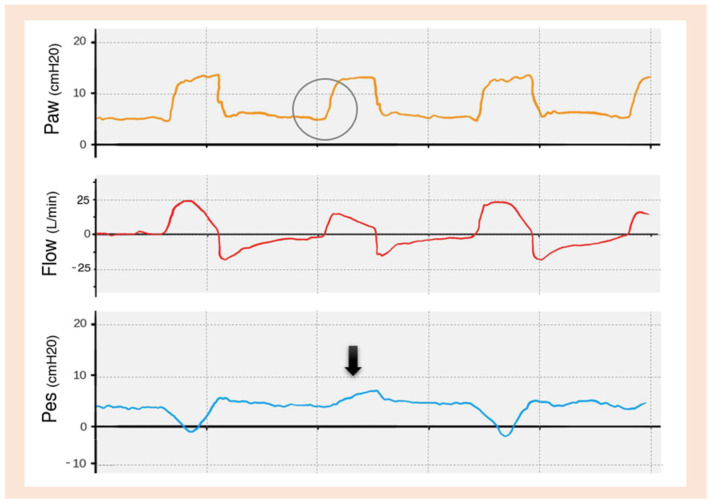
Autotriggera—a high degree of clinical suspicion is required to detect the presence of autotrigger. The presence of mechanical breaths without a previous effort by the patient, i.e., with the absence of negative deflection in the pressure (circled area) and the Pes (small arrow) waveforms, strongly suggests it. Its occurrence simultaneous with the patient’s heart rate may indicate strong cardiac oscillations as one of the possible causes. Pes: esophageal pressure.

Identifying this PVA may be challenging since the patient’s discomfort may be very unspecific. In the case of artifacts in the ventilatory graphs, as depicted in Figure 3, discrete irregularities may be noted; specifically, if caused by intense cardiac oscillations, these irregularities will coincide with the patient’s heart rate [18]. Also, the maximum inspiratory flow will be inferior to the flow achieved with patient-triggered breaths. Moreover, in this PVA, there will be no negative deflection in the pressure waveform before the mechanical breath is delivered [23]. This latter phenomenon is even more evident if an esophageal catheter is in place, displaying the complete absence of patient effort [28].

Managing autotrigger begins with the inspection of the ventilator circuit for any fluid collections or air leaks. Then, the trigger sensitivity should be reduced steadily, always ensuring that all of the patient’s efforts are still detected [16,25,29]. An alternate solution may be to shift to a pressure-trigger [25,29].

##### Double Trigger

This clinical situation is observed when two consecutive mechanical breaths are provided after only one patient’s inspiratory effort is made. It is very frequent and may originate significant clinical implications, with excessive tidal volumes [16], predisposing the patient to ventilator-induced lung injury (VILI) [17]. The patient will show signs of discomfort and cough. Once again, the physician may approach the patient and enquire if the ventilator provided two successive mechanical breaths.

**Figure 4:**
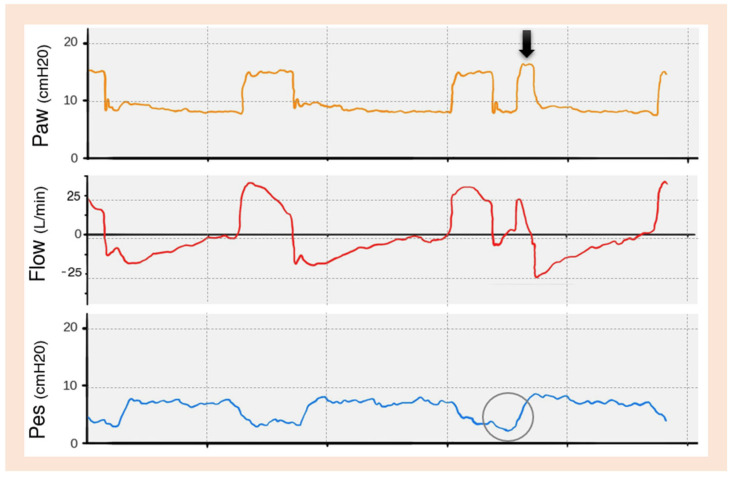
Double trigger—Two consecutive mechanical breaths will be present, albeit they will present certain dissimilarities—in the second breath, the decrease in airway pressure after the inspiratory phase will occur faster than in the initial breath (arrow). The Pes tracing will demonstrate the patient is performing only a prolonged inspiratory effort (negative deflection), that persists and originates the second triggering of the ventilator (circled area). Pes: esophageal pressure.

The ventilator waveforms will show that in the pressure scalar after the inspiratory phase has ceased, the loss of airway pressure is much more abrupt than expected when compared to the previous breath. Even more clearly, there will be two consecutive breaths observed. Furthermore, if an esophageal pressure monitoring curve is available, an inspiratory effort by the patient (negative deflection) will be present throughout both of the mechanical breaths delivered, thus demonstrating the strenuous effort causing the double triggering of the ventilator, as highlighted in Figure 4.

Sources for this PVA are increased respiratory drive, early cycling, reduced inspiratory pressure support, and coughing [18,25]. Patients with an elevated respiratory drive (fever, anxiety, pain, etc.) exert a very intense and prolonged inspiratory effort, resulting in an additional mechanical breath even before the complete expiration of the first breath has ceased. Subsequently, the second breath will be “stacked” on the first one; hence, this PVA may also be referred to as “breath stacking”. This may occur simply because the inspiratory pressure support is insufficient to the patient’s momentaneous (or sometimes not so short) demands.

Early cycling is another possible cause of double triggering. In this case, the patient’s neural inspiratory time exceeds the ventilator inspiratory time, and consequently, the patient’s inspiratory effort continues even though the ventilator has closed the inspiratory valve and transitioned to the expiratory phase; here, the effort is powerful enough to trigger the ventilator once more and deliver a second mechanical breath immediately after the first. This will be further discussed in the corresponding section below.

Addressing the root of the patient’s increased inspiratory effort is essential—managing pain, fever, or anxiety are crucial steps. If the patient requires more inspiratory flow without the need for an overtly excessive tidal volume, providing higher pressure support may be adequate. Nevertheless, if the problem resides in early cycling, then the cycling parameter should be, in this case, thoroughly decreased (e.g., from 30% to 25% or less) until the patient is more comfortable [21].

##### Reverse Trigger

The reverse trigger is a recently discovered [32] PVA, clearly defined and separated from the other types of PVAs, notwithstanding the mystery surrounding the main causes and mechanisms. In general terms, reverse triggering takes place in profoundly sedated patients without neuromuscular blockade when a programmed ventilator breath leads to a reflex inspiratory diaphragmatic contraction by the patient, which in turn may trigger the ventilator to deliver a second mechanical breath [26]. 

There are many different proposed pathophysiological mechanisms [33]. One of them entails the stimulation of the diaphragm during the inspiratory phase of the programmed mechanical breath, which then initiates the remainder of the process described above, culminating in a double trigger and breath stacking. This is clinically challenging to detect [18,26]. Since this anomaly occurs in sedated or comatose patients, the physician is unable to obtain information by interviewing the patient. Accordingly, the physician must attempt to distinguish whether the first mechanical breath is patient or ventilator-elicited, where the first scenario is probably related to premature cycling and the latter to reverse triggering. Here, there will be no negative deflection in the pressure scalar preceding the first breath. Moreover, in the second scenario, by placing the hand on the patient’s upper abdomen, the physician may perceive the contraction of the diaphragm occurring just after the first mechanical breath or even while performing an extended expiratory hold maneuver, uncovering any patient’s effort.

**Figure 5:**
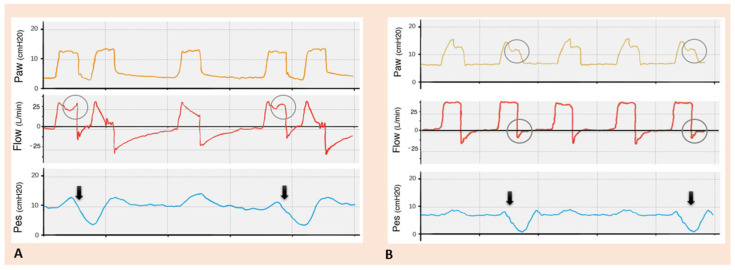
Reverse trigger—This PVA, unlike the others, is present only in deeply sedated patients under controlled mechanical modes. (**A**) In pressure control mode, a small increase in the flow waveform during the deceleration period of the inspiratory phase (circled areas), simultaneous to a small decrease in the pressure waveform, all explained by the diaphragmatic contraction. The Pes graph is essential to highlight the negative swings occurring after the initial mechanical breath (arrows). (**B**) In volume control mode, the mechanical breath will demonstrate reduced peak airway inspiratory pressure and total expiratory flow (circled areas). Once again, the negative deflections in Pes after the first breath are present (arrows). Pes: esophageal pressure; PVA: patient–ventilator asynchrony.

Furthermore, other changes in the ventilator waveforms may be noted, as indicated in Figure 5. In general terms, there will be a reduced peak expiratory flow in the affected breaths [26]. Other changes will vary depending on the ventilatory mode applied to the patient. If the mode is pressure controlled (Figure 5A), one may detect a minor increase in the flow scalar during the deceleration part of the inspiratory phase, along with a slight decrease in pressure due to the contraction of the diaphragm. On the other hand, if a volume-controlled mode is employed (Figure 5B), both the peak airway inspiratory pressure and the total expiratory flow will be reduced. If the contraction induced by the first mechanical breath is vigorous enough may induce the triggering of a second breath, with consequent breath stacking [33].

The analysis of the esophageal pressure graph may aid by demonstrating repetitive negative swings in Pes occurring after the beginning of the initial mechanical breath (that conveys a passive rise in Pes), thus indicating the presence of reverse triggering [20,25,33].

Proposed solutions include reducing the sedation or reducing the programmed mandatory respiratory rate, both targeting the patient to become more independent from the ventilator; on the other hand, if refractory cases appear, vigorous efforts, breath stacking, and increased tidal volumes with possible VILI, and the initiation of neuromuscular blockade are advised [16,18].

#### 5.1.2. Cycling

##### Early Cycling

Regarding the transition of the inspiratory to the expiratory phase, early cycling takes place when the duration of the mechanical inspiratory phase is inferior to that of the patient’s neural inspiratory effort [26,27]. This phenomenon is also called premature or short cycling. The patient will seem uncomfortable and agitated, tachypneic, and enduring a strenuous effort during the inspiratory phase.

**Figure 6:**
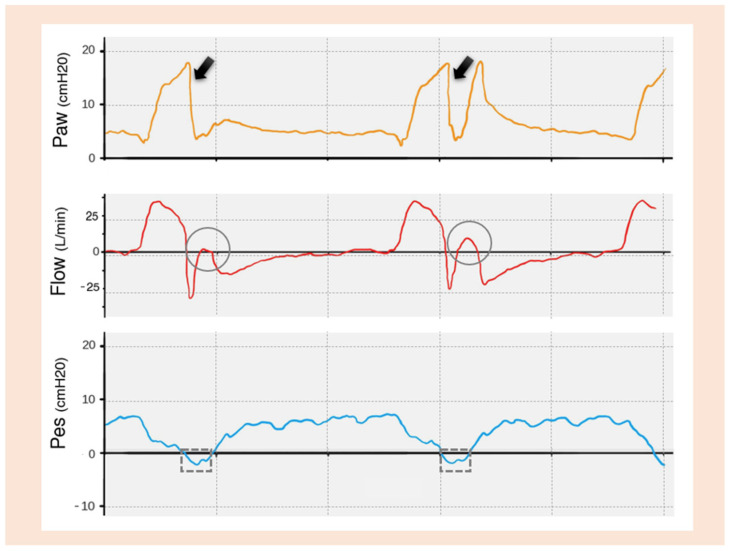
Early cycling—The patient’s inspiratory neural time exceeds the ventilator inspiratory time, mainly leading to distortion with an initial upward deflection of the expiratory flow waveform (circled areas). Furthermore, the deceleration after inspiration will be accelerated, with a near-vertical drop in the airway pressure waveform (arrows). In the Pes waveform, the patient’s effort persisting throughout inspiration and expiration, is unmasked (dashed line square). Should this asynchrony be strenuous enough, a phenomenon of double-trigger with breathstacking may occur (see second respiratory cycle). Pes: esophageal pressure.

As illustrated in Figure 6, the ventilator waveforms present a near-normal inspiratory flow wave, followed by a distortion of the expiratory flow wave, characterized by an initial upward deflection [23,24]. This is explained by the patient’s maintained inspiratory effort with respiratory muscle contraction [31]. Furthermore, compared to the previous unaltered breaths, the inspiratory phase is shorter and the maximum expiratory flow will be lower. Also, the decelerating phase after inspiration will be less gradual, appearing with a near-vertical drop on the pressure curve.

If vigorous enough, this additional effort may indeed generate a double trigger with breath stacking [18,26,27]. Moreover, in the Pes tracing, a negative deflection reflecting the patient’s active inspiratory effort will persist during the expiratory phase [18,20].

The main causes attributed to premature cycling consist of excessive inspiratory efforts by the patient, inadequately short cycling parameters (a high % of maximum inspiratory flow, in the case of pressure support), or brief inspiratory machine time (in volume-controlled and pressure-controlled ventilation modes) [18,25].

Managing this asynchrony involves controlling the respiratory rate or prolonging the duration of the inspiratory phase either by reducing the cycling parameter (i.e., decreasing the % of maximum inspiratory flow) in pressure support mode [4,21,29] or by increasing the inspiratory time in controlled mandatory modes [25,29].

##### Late Cycling

On the other side of the spectrum lies late or delayed cycling, where the mechanical insufflation time exceeds the patient’s neural inspiratory phase onto the expiratory phase [23,25].

The patient may indicate that the breath does not terminate appropriately; also, the patient may display an active expiratory muscle contraction suggestive of expiration [17] when the ventilator is still delivering the breath [27].

**Figure 7:**
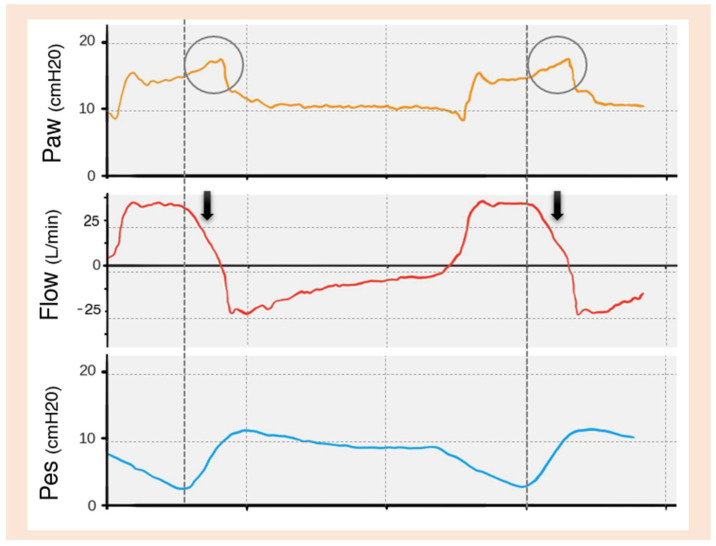
Late cycling—The flow scalar demonstrates a shift from a more gradual to a more rapid decrease in inspiratory flow (arrow). Also, more characteristically, the pressure waveform depicts a positive deflection at the end of inspiration (circled area), corresponding to the patient’s active expiratory effort against a closed expiratory valve since the ventilator is still providing inspiratory support. The esophageal catheter waveform demonstrates that the patient’s neural inspiratory time has ceased, even though the mechanical ventilator inspiratory time clearly exceeds it, causing a positive deflection in this curve (dashed line).

The ventilator waveform is relatively straightforward—in the pressure scalar, at the end of inspiration, a positive inflection will be observed, explained by the active effort of the patient of exhalation against a closed expiratory valve that could register a pressure above the set pressure support (the ventilator is still delivering a mechanical breath) [16,25,26,29]. Simultaneously, there will be a change from a gradual to a more rapid decrease in inspiratory flow, observed in the flow scalar [17,23,29]. Moreover, the esophageal catheter will demonstrate that the patient’s effort has ceased (the corresponding negative swing in Pes stops), even though the ventilator is still providing active assistance (with a progressive rise in Pes) [20]. All of these changes are presented in Figure 7.

Causes comprise a prolonged programmed inspiratory time (in pressure-controlled mandatory ventilation) or a late cycling parameter (very low % of maximum inspiratory flow in pressure support ventilation).

Similarly to early cycling, late cycling presents a rather forthright approach, including either reducing the inspiratory time in controlled mandatory modes or shortening the breath by increasing the % of maximum inspiratory flow (e.g., from 30% to 35%) [25].

#### 5.1.3. Flow

##### Flow Overshoot

Flow overshoot occurs when the inspiratory flow exceeds the needs of the patient. This may give rise to a short inspiratory time and an elevated airway pressure at the beginning of the inspiratory phase. Although this constitutes an uncommon phenomenon, flow overshoot may negatively impact the patient, namely discomfort, and may alter the cycling off to the expiratory phase—by creating a “new” exaggerated peak inspiratory flow, the ventilator will consequently transition to the expiration earlier, at a higher % of the peak flow. If maintained, this may produce an early cycling phenomenon and air hunger.

At the bedside, the patient will be uncomfortable and may refer to a surplus in pressure in inspiration and periods of tachypnea. Sources of this PVA include increased assisted pressure or a short set rise time, which consequently originate a sharp increase in inspiratory pressure and flow.

**Figure 8:**
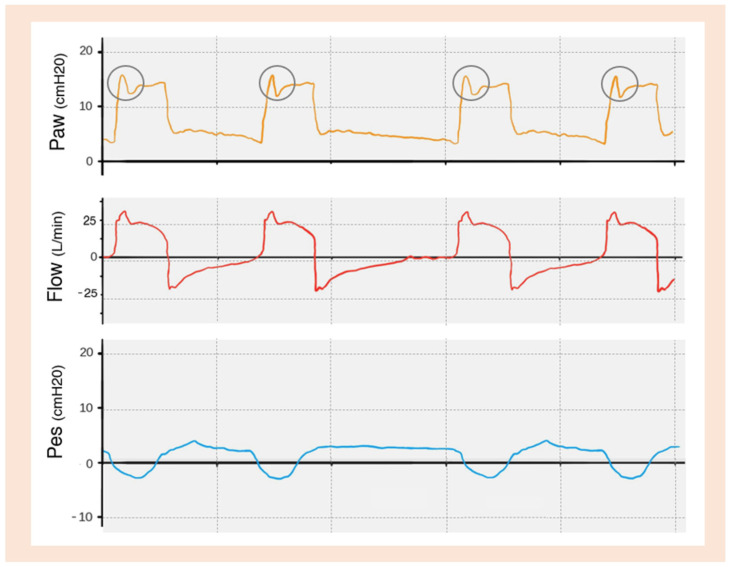
Flow overshoot—The excessive inspiratory flow creates a steep initial peak in the airway pressure waveform (circled areas), and the inspiratory phase may be shortened.

The ventilator curves are very characteristic, where an initial elevation in the pressure graph is observed [17], and the total duration of the inspiratory phase may be diminished, which may be visualized in Figure 8. A very steep inspiratory ramp is almost always present.

Managing flow overshoot involves prolonging the duration of rise time so that the start of inspiration occurs more slowly, adjusting this to the patient’s “air hunger”. Another option is to reduce the programmed assisted inspiratory pressure, thus leading to a more adequate inspiratory flow [25].

##### Flow Starvation

This type of asynchrony takes place when the provided inspiratory flow is insufficient to the patient’s demands [31]. The patient will demonstrate discomfort, exerting continuous effort for further pressure and assistance. Tachypnea and paradoxical thoracoabdominal movements may be observed.

This exaggerated effort, if continuous and progressive, exposes the patient to lung injury due to the excessive negative pleural pressures and consequent high transpulmonary pressures [27]. Furthermore, this effort will generate an additional energy expenditure due to respiratory muscle usage, thus further limiting the ventilator weaning process overall, possibly contributing to extubation failure.

**Figure 9:**
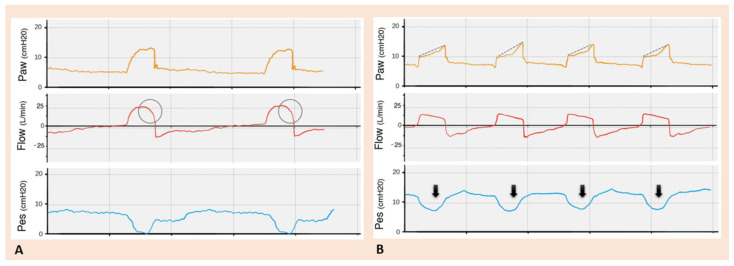
Flow starvation—(**A**) In assisted-pressure mode, the flow curve will have a rounded shape (circled area), contrasting with the generally triangular shape in patients without any PVA. (**B**) In volume-assisted mode, the airway pressure waveform will present a concave shape, which may be progressively more pronounced depending on the patient’s effort (dashed line). Once again, the Pes waveform will help by identifying very negative swings during the inspiratory phase, consequent to the patient’s vigorous efforts (arrows) due to inadequate flow. Pes: esophageal pressure; PVA: patient–ventilator asynchrony.

Regarding the ventilator waveforms, depending on the ventilatory modality, distinct alterations may be observed. In assisted pressure modes, the inspiratory flow curve will present a rounded shape (versus a triangular shape in the absence of a PVA) (as in Figure 9A) [23], whilst, in volume-assist, the pressure scalar graph will become increasingly negative with a concave shape, parallel to the patient’s progressive effort (as demonstrated in Figure 9B) [17,28,31]. This last waveform may mirror the situation when patients present tidal volumes that overcome their lung compliance (known as increased stress index >1) [34]; nevertheless, this scenario occurs in patients under mandatory volume-controlled modes, whereas flow starvation requires an effective active inspiratory effort [25]. In Pes tracing, the patient’s efforts will be identified as negative swings during the inspiratory phase [20].

The main causes of flow starvation include reduced or inadequate assisted pressure/volume or an inadequately prolonged rise time in the presence of “air hunger”.

Managing this PVA is relatively straightforward, either by providing the patient with further inspiratory support [23] or shortening the rise time according to the patient’s air hunger [21]. If, despite these measures, the asynchrony does not improve and the patient’s effort is excessive and related to another external factor (pain, agitation, others), one may consider lightly augmenting the sedation.

## 6. Conclusions

Patient–ventilator asynchronies (PVAs) are a prevalent and significant challenge in the management of ventilated patients in the ICU, often leading to adverse clinical outcomes. While numerous diagnostic tools are available, bedside clinical examination and vigilant monitoring of ventilator waveforms remain fundamental yet complex approaches to identifying and addressing PVAs. Optimizing ventilator settings to match the specific asynchrony remains the most effective intervention. To ensure optimal patient care, continuous education, and hands-on practice in waveform interpretation are essential, enhancing clinical expertise and fostering improved outcomes in critical care settings.

## Figures and Tables

**Table 1:** Classification of patient–ventilator asynchronies.

**TRIGGERING**	Inspiratory trigger delay
	Ineffective trigger
	Autotrigger
	Double trigger
	Reverse trigger
**CYCLING**	Early cycling
	Late cycling
**FLOW**	Flow overshoot
	Flow starvation

## Data Availability

Not applicable.
